# Suicide rates and risk factors for suicide among Israeli immigrants from Ethiopia (1985–2017)

**DOI:** 10.1186/s13584-021-00454-0

**Published:** 2021-03-23

**Authors:** Rafael Youngmann, Nelly Zilber, Ziona Haklai, Nehama Goldberger

**Affiliations:** 1 Clinical Psychology Department, Ruppin Academic Center, Emek Hefer, Israel; 2 The Falk Institute for Mental Health Studies, Kfar Shaul Hospital, Jerusalem, Israel; 3 Division of Health Information, Ministry of Health, Jerusalem, Israel

**Keywords:** Suicide, Risk factors, Ethiopian immigrants, Sociocultural background

## Abstract

**Background:**

Suicide rates among Ethiopian immigrants to Israel (EI) are relatively high. This study sought to identify suicide-risk factors in this population in order to suggest some potentially preventive measures to mental health policymakers who are struggling to prevent suicide among EI.

**Method:**

Nationwide age-adjusted suicide rates were calculated for EI, Former Soviet Union immigrants (FSUI) and Israeli-born (IB) Jews by age, gender, and year of death and, for EI, by marital status and immigration period in the years 1985–2017 (1990–2017 for FSUI).

**Results:**

Age-adjusted suicide rates for the period 1990–2017 confirmed the significantly higher rate among EI––3.1 times higher than for FSUI and 4.1 times higher than for IB. Similar rates were obtained for both genders, within each age group, and in all study years. Comparable male/female rate ratios were found among EI and IB (3.3, 3.6, respectively). Over the years of the study, only among the Ethiopian immigrants were there large fluctuations in suicide rates: a decrease (1992–2001), followed by an increase (2001–2006), and then a progressive decrease (from 2006). The secular changes differed greatly according to age. Among females, these fluctuations were smaller, the decrease began earlier and was greater, and the subsequent increase was much smaller. Marriage was found to be less protective for Ethiopian immigrants than for the other surveyed populations.

**Conclusions:**

The considerable gap between the EI’s and FSUI’s suicide rates highlights the critical role of immigrants’ integration difficulties. These difficulties among EI lead to ongoing conflict within the family, which may explain why marriage is less protective for EI. Nevertheless, progressive integration is occurring as indicated by the decline in suicide rates since 2006. The fluctuations in EI suicide rates over time seem to be associated with modifications in social welfare allowances, which are crucial for EI of low socioeconomic status. Groups at risk, particularly EI men facing socioeconomic challenges and EI with considerable family conflict, typically identified by HMOs and welfare services, should be screened for suicide risk, and those identified as at risk referred to tailored workshops sensitive to Ethiopian culture.

## Introduction

The prevailing cultural ethos in most societies sanctifies human life and thus rejects suicide, but the incidence of suicide is high worldwide: In 2016, suicide accounted for 1.4% of all deaths and was the 18th leading cause of death worldwide [1].

Suicide rates vary in different countries and in different ethnic or religious populations, even within a given country [2]. In Israel, for instance, in 2006–2008 suicide rates among European- or American-born immigrants aged 45 years or more were higher than among Asian- or North African-born [3]. Already in the 1970’s immigrants’ suicide rates were reported higher overall than those born in Israel among the elderly population (aged over 55) [4].

Immigration, with its uprooting experience and acculturation-derived stress, has been repeatedly reported to be a risk factor for mental disorders, notably, depression disorders, which in turn, comprises a risk factor for suicide [5–7]. However, the link between immigration and suicide risk is not unequivocal. Among many immigrant populations, for both sexes, the suicide rate in their new country has been reported to be higher than among the native-born (e.g., [8, 9]). In other cases [8, 10], the opposite trend was observed, whereas in still other studies, it was found that in a given country, some immigrants presented higher suicide rates and others lower rates than non-immigrants (e.g., [11]).

These differences in immigrant suicide rates might be better understood by comparing culturally different populations in a given country. Israel presents a unique opportunity for such a comparison, given that about a quarter of Israel’s Jewish population was born abroad, arriving from various countries with marked cultural differences [12]. Moreover, assistance for absorption into the country is provided to all Jewish immigrants, independent of their origin: Israel’s Law of Return entitles Jews living anywhere to immigrate to Israel and receive, among other benefits, immediate Israeli citizenship, free medical care, and language training.

In recent decades, two large groups of immigrants, characterized by marked cultural differences, immigrated to Israel––from the Former Soviet Union and from Ethiopia. Of the larger group, Former Soviet Union immigrants (FSUI), more than half a million immigrants arrived between 1990 and 1995––a third arrived in 1990–1991, and about half arrived throughout the 1990s [13].

The next largest group of immigrants arrived from Ethiopia. At the end of 2017, the population of Israeli citizens of Ethiopian origin numbered 148,700, of whom 87,000 (59%) were born in Ethiopia [14]. This population can be divided into three groups who came to Israel under very different circumstances. About a quarter of Ethiopian immigrants (EI) came before 1991 (*Operation Moses*), having fled on foot from Ethiopia to Sudan, where they waited in refugee camps for long periods to be rescued. Another quarter of the EI arrived in 1991 (*Operation Solomon*), most of them having been airlifted directly from Addis Ababa in May 1991. EI arriving after 1991 made the trip alone or in small groups on regular flights from Addis Ababa [14, 15].

The cultural differences between immigrants from Russia and those from Ethiopia are self-evident. Most EI previously lived as peasants and artisans in the Ethiopian highlands [15], and in Israel, they generally comprise a low-skilled population. In contrast, FSUI are typically highly skilled, with a high proportion of college graduates [16].

Although the suicide rate for both groups of immigrants has been described as higher than for IB, the EI rate was especially high and alarming [17]. The present study seeks to enhance our understanding of the high suicide rate among EI in order to suggest some potential preventive measures to mental health policymakers who are struggling to prevent suicide among EI. To this end, we analyzed suicide rates among EI by age, gender, year of death, marital status, and immigration period, in comparison with suicide rates among immigrants from another country (FSU) and the Israeli-born population.

## Methods

### Research population

The research population comprised three groups, aged 15 and over: All EI who had immigrated since 1980, all FSUI who had immigrated since 1990, and IB Jews.

### Data collection

Suicide rates were calculated for the period 1985–2017 for EI and IB. FSUI suicide rates were calculated for the period 1990–2017, as relatively few FSU immigrants arrived between 1985 and 1989.

Data on suicides were obtained from the nationwide database of causes of death, maintained by Israel’s Central Bureau of Statistics (CBS), who collect data from death certificates, supplemented by other sources, primarily from the Institute of Forensic Medicine.

The denominator population used for rate calculation in each group was the cumulative mid-year populations for each period, by age, calculated from the Annual Statistical Abstracts of the CBS and their other publications on Ethiopian immigrants [18, 19]. These documents were supplemented by additional data the CBS calculated for the current study, including EI population by calendar year and by marital status.

### Data analysis

As the age distribution was very different in the three research populations and with suicide rates varying with age, age-adjusted suicide rates were calculated. Direct standardization was used, with the standard population being the total Israeli population of 1996 in four age groups––15–24, 25–44, 45–64, and 65 and above.

As some groups had relatively few suicides, we calculated 5-year running average rates, computed from the number of suicides over each 5-year period, divided by the cumulative population for these years. The corresponding rates were assigned to the mid-year of each period.

The age-adjusted rates were calculated by age, gender, and year of death, and, for EI, by marital status and immigration period. Rates were based on at least 20 cases, except for rates by year of death among EI females, the latter being based on at least 10 cases. Rate ratios (RR) and 95% confidence intervals (CI) for the rates and rate ratios were also calculated. Where the CI of the RR did not include 1.0, the rates were considered significantly different. The data analysis was done with SAS 9.2, with further calculations in excel.

## Results

During the study period of 1990–2017, 447 EI (353 men, 94 women), 2116 FSUI (1672 men, 444 women), and 4194 IB (3324 men, 870 women) committed suicide. Age-adjusted suicide rates for this period were significantly higher among EI than among the other two populations: 2.5 times higher than for FSUI and 4.1 times higher than for IB, who had the lowest rates of the study populations (Table 1). Similar results were obtained for both men and women and within each age group (Tables 1 and 2).

**Table 1:** Age-adjusted suicide rates per 100,000 population (age 15^+^) for the three population groups (1990–20(17, and rate ratios (95% confidence intervals are indicated in parentheses)

	Ethiopian immigrants (EI)		Former Soviet Union immigrants (FSUI)		Israeli-born (IB)		Rate ratio		
	*N*	Rate	*N*	Rate	*N*	Rate	EI/FSUI	EI/IB	FSUI/IB
Total	447	30.2 (27.4–33.1)	2116	12.0 (11.4–12.5)	4194	7.3 (7.1–7.6)	2.5 (2.3–2.8)	4.1 (3.7–4.6)	1.6 (1.5–1.7)
Males	353	47.5 (42.5–52.6)	1672	21.1 (20.0–22.1)	3324	11.5 (11.1–12.0)	2.3 (2.0–2.5)	4.1 (3.7–4.6)	1.8 (1.7–1.9)
Females	94	13.0 (10.4–16.0)	444	4.2 (3.8–4.6)	870	3.2 (2.9–3.4)	3.1 (2.4–3.9)	4.1 (3.3–5.1)	1.3 (1.2–1.5)
Rate ratio (males/females)		3.7 (2.9–4.6)		5.0 (4.5–5.6)		3.6 (3.3–4.0)			

*N* Number of suicides

**Table 2:** Age-specific suicide rates per 100,000 population for the three population groups (1990–2017) and rate ratios (95% confidence intervals are indicated in parentheses)

Age (years)	Ethiopian immigrants (EI)		Former Soviet Union immigrants (FSUI)		Israeli-born (IB)
	Rates	Rate ratio (EI/IB)	Rates	Rate ratio (FSUI/IB)	Rates
A. Both genders					
15–24	31.7 (26.5–37.6)	6.2 (5.1–7.4)	10.6 (9.3–11.9)	2.1 (2.4–3.7)	5.1 (4.8–5.5)
25–44	28.0 (24.1–32.4)	4.5 (3.8–5.2)	12.1 (11.3–13.0)	1.9 (2.0–2.7)	6.3 (6.0–6.6)
45–64	32.4 (26.1–39.9)	3.6 (2.9–4.5)	11.9 (11.0–12.9)	1.3 (2.2–3.4)	9.0 (8.4–9.5)
65^+^	29.8 (21.7–40.0)	2.7 (1.9–3.7)	14.3 (13.1–15.6)	1.3 (1.5–2.9)	11.2 (9.9–12.6)
Rate ratio (65^+^/15–24)	0.9 (0.7–1.3)	–	1.4 (1.2–1.6)	–	2.2 (1.9–2.5)
B. Males					
15–24	53.3 (43.9–64.1)	6.6 (5.4–8.1)	18.0 (15.7–20.5)	2.2 (2.4–3.7)	8.1 (7.5–8.7)
25–44	44.2 (37.2–52.1)	4.5 (3.7–5.3)	21.5 (19.9–23.3)	2.2 (1.7–2.5)	9.9 (9.4–10.4)
45–64	49.5 (38.4–62.9)	3.5 (2.7–4.5)	21.1 (19.2–23.0)	1.5 (1.8–3.1)	14.3 (13.4–15.3)
65^+^	43.1 (29.5–60.8)	2.5 (1.7–3.7)	25.3 (22.7–28.1)	1.5 (1.2–2.5)	17.3 (15.0–19.9)
Rate ratio (65^+^/15–24)	0.8 (0.5–1.2)	–	1.4 (1.2–1.7)	–	2.1 (1.8–2.5)

(there were insufficient cases to make the analysis among females)

Suicide rates in all groups were significantly higher for men than for women, particularly among FSUI (male/female rate ratio of 3.7 and 3.6 among EI and IB respectively, vs. 5.0 among FSUI; Table 1). The EI/IB ratio was comparable for both genders, whereas, for EI/ FSUI, it was significantly lower for males than for females.

For EI, the age-specific rates were similar at all ages, whereas for IB the rates increased with age, with a significant increase from one age group to the next one; the same was true for FSUI, but with a smaller increase and similar rates at ages 25–44 and 45–64 (rate ratio between the oldest and the youngest 1.4 for FSJU and 2.2 for IB). Consequently, the rate ratio (RR) for immigrants compared with IB decreased with age, particularly for EI (Table 2). Similar results were observed among males (there were not enough cases to do the analysis among women).

For EI, the age-specific suicide rates were comparable at all ages, whereas, for IB, the rates increased with age, with each age group reflecting a significant increase over the younger group; the general trend was similar for FSUI, but with smaller increases with age and similar rates at ages 25–44 and 45–64 (the rate ratios between the oldest and the youngest age categories were 1.4 for FSJU and 2.2 for IB). Consequently, the rate ratio (RR) for immigrants, compared with IB, decreased with age, particularly for EI (see Table 2). Similar results were observed among males (there were insufficient cases to analyze women).

In order to understand why the EI suicide rate did not increase with age, we compared age-specific rates for EI during three periods: Period 1 (1985–2000), which was the closest to the large immigration waves; Period 2 (2001–2010), during the 10 years following their immigration; and Period 3 (2011–2017), during the last years of the study (Table 3). Among the 15–24-year-olds, the suicide rate doubled in Period 2, but in Period 3, it diminished to the Period 1 level. In the 25–44 age group, the suicide rate was more stable, but it also tended to diminish in Period 3. Finally, among the 45–64-year-olds, the rate was significantly higher in Period 1 than in the two subsequent periods, for which the rates were comparable. Thus, the EI suicide rate was the highest for the youngest age group in Period 2, and highest for the 45–64 age group in Period 1. In Period 1, the rate increased with age, whereas the youngest age group had the highest rate in Period 2. In Period 3, the rates were comparable at all ages, with slight increases with age. Thus, in Period 3, rates for all EI age groups were lower than in Period 1.

**Table 3:** Age-specific suicide rates per 100,000 population for Ethiopian immigrants in three periods of the study and rate ratios (95% confidence intervals are indicated in parentheses)

	Period	Age (years)		
		15–24	25–44	45–64
Total	1985–2000	22.8 (15.8–31.8)	30.6 (22.3–41.0)	51.4 (35.6–71.9)
	2001–2010	44.8 (35.6–55.7)	31.3 (24.9–38.9)	29.4 (20.0–41.8)
	2011–2017	20.7 (13.0–31.4)	22.8 (17.4–29.4)	26.5 (17.9–37.8)
Rate ratio (1985–2000 / 2001–2010)		0.5 (0.3–0.8)	1.0 (0.7–1.4)	1.7 (1.0–2.9)
Rate ratio (1985–2000 / 2011–2017)		1.1 (0.5–2.5)	1.3 (0.9–2.0)	1.9 (1.2–3.3)
Males	1985–2000	37.3 (24.8–53.8)	36.6 (24.1–53.2)	72.8 (46.7–108.4)
	2001–2010	75.7 (59.1–95.5)	53.3 (41.3–67.7)	41.5 (25.7–63.5)
	2011–2017	[33.3 (19.7–52.6)]	37.7 (27.9–49.9)	45.5 (29.4–67.1)
Rate ratio (1985–2000 / 2001–2010)		0.5 (0.3–0.8)	0.7 (0.4–1.1)	1.8 (0.9–3.3)
Rate ratio (1985–2000 / 2011–2017)		[1.1 (0.6–2.1)]	1.0 (0.6–1.6)	1.6 (0.9–2.9)

*Note.* Values in brackets are based on fewer than 20 cases

(there were insufficient cases to make the analysis among females)

**Fig. 1:**
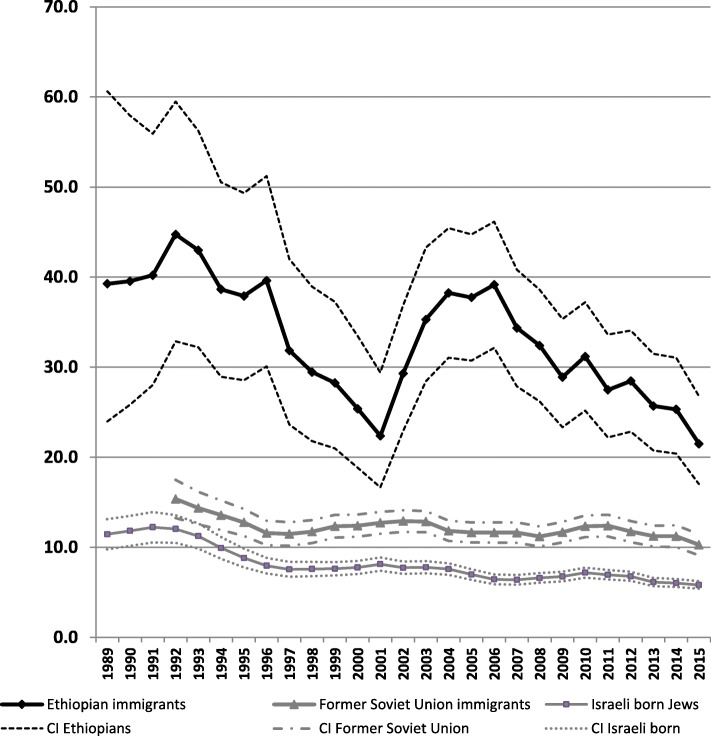
Secular trend of the age-adjusted suicide rates among Ethiopian immigrants, Former Soviet Union immigrants and Israeli-born. Rates per 100,000 population with 95% confidence interval (CI)

We compared the secular trend of age-adjusted suicide rates for EI, FSUI, and IB between 1985 and 2017 for EI and IB, and between 1990 and 2017 for FSUI (Fig. 1). For the entire period, suicide rates were significantly higher for EI than for FSUI and IB and lowest for IB, with FSUI rates ranging between 1.3 and 1.8 times higher than the IB rates. Some decrease over the years was observed in the three population groups. Among EI the rate in 2015 was about half the rate in 1992, a similar decrease as among IB, but among EI, contrary to the other populations, there were substantial secular changes: The EI rate decreased from 1990 until 2001, increased till 2006, and then decreased again. Similar trends were observed for FSUI and IB, but among EI, the changes were much greater (e.g., 75% for the second increase vs. 8% among IB, and 10% among FSUI). Moreover, the fluctuations began later: The first decrease ceased in 1997 for IB and FSUI and only in 2001 for EI, and the second decrease began around 2003 for IB and FSUI, and only in 2006, for EI.

**Fig. 2:**
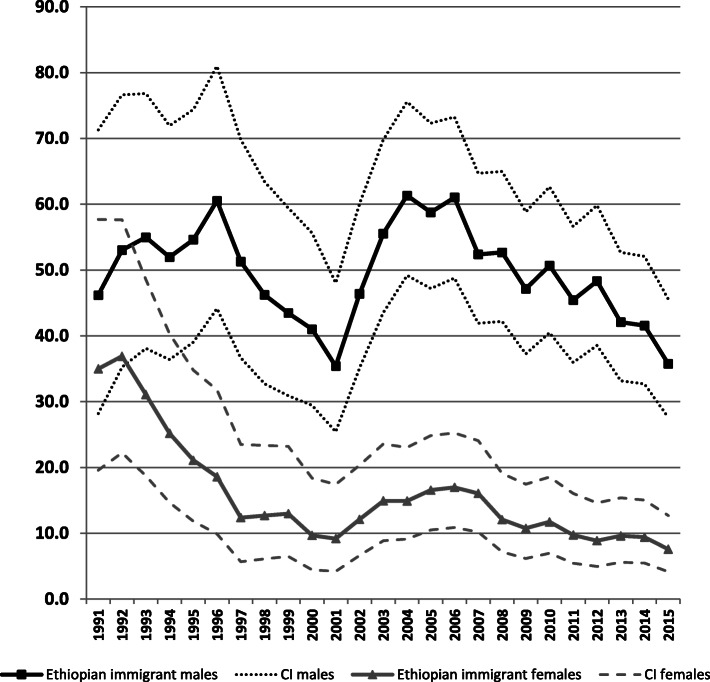
Secular trend of the age-adjusted suicide rates among Ethiopian immigrants, by gender. Rates per 100,000 population with 95% confidence interval. Rates for males based on 20 or more cases and for females on 10 or more cases. Rates are 5-year running averages (the number of suicides over each 5-year period divided by the cumulative population for these years). The year on the axis of the figure is the mid-year of each 5-year period

As the EI’s suicide rates and secular trends significantly differed from those of the two other groups, we performed additional analyses, targeting the EI population***.*** Upon examining secular trends by gender (Fig. 2), we found lower rates for females in all the examined years, but only from 1995 did this trend reach significance. Suicide rates fluctuated over the years and these fluctuations were smaller among females. Both genders were characterized by a decrease, followed by an increase, and then a progressive decrease in suicide rates. However, rates began to decrease earlier for women (beginning from 1993) than for men (beginning from 1997). Moreover, the decrease was greater for women, and the subsequent increase more limited; EI men’s suicide rates, after 2001, returned to their values before the downturn, while rates for women remained low.

To examine whether the substantial secular changes in age-adjusted rates for EI might have been related to different migration dates and circumstances, we compared the three waves of Ethiopian immigration to Israel (before 1991, during 1991, and after 1991) and found similar secular trends.

We also examined suicide rates among 25–64-year-old EI by marital status during 1996–2001, the period for which population data were available. Suicide rates were the lowest for married EI, as was the case for the general population. The RR (suicide rate for EI / suicide rate in the general population, excluding EI) was highest for married, lower for single, and lowest for divorced or widowed (Table 4). Similar results were found for both genders.

**Table 4:** Age-adjusted suicide rates per 100,000 population by marital status (age 25–64) for Ethiopian immigrants and for the general population excluding Ethiopian immigrants (1996–2011) and rate ratios (95% confidence intervals are indicated in parentheses)

Marital Status	Ethiopian immigrants (EI)	General population (GP)	Rate Ratio (EI/GP)
Married	22.7 (17.9–28.4)	4.3 (4.1–4.5)	5.3 (4.1–6.7)
Single	47.4 (34.9–62.8)	15.3 (14.4–16.2)	3.1 (2.3–4.2)
D-W.^a^	36.3 (24.3–52.2)	16.7 (15.6–17.9)	2.2 (1.5–3.2)
Rate ratio Single/Married	2.1 (1.4–3.0)	3.5 (3.3–3.8)	
Rate ratio Single/D-W*	1.3 (0.8–2.1)	0.9 (0.8–1.0)	

^a^*D-W* Divorced or widowed

## Discussion

The current study presents national suicide rates by various factors among immigrants from Ethiopia, as compared with immigrants from the FSU (characterized by a very different sociocultural background), and to the Israeli-born population. Spanning 33 years, despite wide fluctuations, the suicide rate remained much higher among EI than among the other examined populations. A suicide rate higher than in the local population was also found among Ethiopians who had immigrated to London [20]. Before examining risk factors for suicide among EI in order to develop adequate preventive services adapted to this population, we investigated the possibility that these high rates are primarily due to factors unrelated to life in Israel.

*Differences in suicide rates may be an artifact if under-reporting of suicides differs in various populations*. The reporting of suicides among immigrants and non-immigrants has been examined in the Tel Aviv area [21]. They observed no significant difference in under-reporting between Israeli non-immigrants and African-born immigrants, who were almost exclusively EI in the years of the study [22]. The high rate among EI cannot thus be explained by a higher reporting rate in this population. Suicides were found to be reported slightly more frequently for the European-born population, primarily consisting of FSUI during the years of the study [22]. Thus, the disparity found between FSUI and IB might, indeed, be slightly smaller, but our calculations indicate that the Israeli-born population would still have the lowest suicide rate.

*Variable attitudes toward suicide among cultures* have been identified as a critical factor in understanding why suicide rates vary across different cultures. Both in Israel and the FSU, as well as among FSUI, negative attitudes toward suicide have been reported [23–25], and there is no indication that suicide is less stigmatized among FSUI than among IB. Suicide is also considered a grievous sin among the Ethiopian Jews, both in Ethiopia [26] and in Israel [27]. Thus, differences in cultural attitudes toward suicide may be ruled out as a significant factor in explaining our results.

*Suicide rates in the new country could also reflect the rate in the immigrants’ native country*. There is, however, no evidence of elevated suicide rates in Ethiopia, although the rates may be underestimated due to the quality of vital statistics. Medical records from a general hospital in Western Ethiopia reported a mean annual suicide rate of 4.5 per 100,000 inhabitants between 1966 and 1972 [28]. The crude annual suicide rate in Addis Ababa was 7.8 per 100,000 population between 1974 and 1988, a period marked by unrest and war in Ethiopia [29]. Moreover, even according to more recent data [2], the age-adjusted suicide rate in Ethiopia (13.1 in 2000) was found to be much lower than among EI in the present study (33.9). It thus appears that high suicide rates for EI compared with IB cannot be attributed to higher rates in Ethiopia.

On the other hand, the WHO [2] reported that age-adjusted suicide rates per 100,000 population in 2000 for many FSU republics were much higher than for FSUI in Israel (12.0): 35.0 in the Russian Federation, 29.8 in Ukraine, 37.6 in Kazakhstan, and 49.9 in Lithuania It may be that among FSU Jews the rates were somewhat lower than among the general FSU population: It has been reported that the age-standardized mortality rate for an amalgam of homicide, suicide, and unspecified violent death is lower for Jews than for non-Jews [30]. While no data for suicide alone are available, suicide rates are unlikely to be lower for Jews in Russia than for FSUI. Thus, the higher suicide rate among FSUI than among IB cannot be attributed to the immigrants simply transplanting their native country’s suicide risk. Rather, immigration appears to have led to a *decrease* in suicide rate compared with the rates in their native country, or at least not to an increase.

The main risk factors of the high suicide rate among EI in Israel must, therefore, be sought in what transpires in the country of immigration. Several factors have been considered in the literature:

- *The disparity between the culture of origin and the culture of resettlement*; this disparity is much greater for EI than for FSUI, resulting in EI having to endure a complex acculturation process [31]. Palmer [20], in his study on Ethiopian migrants to London, claimed a direct causal link between maladjustment in the immigration country and suicide.
- *Low consumption of mental health services* [32]
- *Weakening of social support* [15]*,* while the latter contributes to suicide prevention [33, 34].

To further investigate the cause for the relatively high suicide rate among EI, the relationship between demographic factors and suicide rates was analyzed in this population. Comparing the EI rates with those of another immigrant population (i.e., FSUI) and of IB aimed to reveal some additional clues.

Suicide rates were higher for men than for women for all groups, ages, and years, as is the case worldwide, with few exceptions (e.g., China [35];). The rate ratio (RR) of male to female suicide rates was similar among EI (3.3) and IB (3.6), both close to the ratio (3.5) reported by WHO for high-income countries [2]; the high RR for FSUI (4.8) may be explained by an especially deleterious use of alcohol among men, as a high level of alcohol consumption is associated with increased suicide risk [36].

The similarity between male/female RR among EI and IB and of EI/IB RR in both genders is particularly interesting in view of the considerable difference of status of women in patriarchal Ethiopian society vs. liberal Israeli society. Youngmann and Shokeid [37] conducted interviews with key informants and in the format of focus groups, in the Ethiopian community concerning the causes of EI suicide and possible preventive methods. Both EI men and women agreed that men felt humiliated and threatened when EI women became involved in economic activities in their new country and benefited more than did men from public social agencies, which are mostly served by female professionals. EI men reported believing that social workers and police encourage women to apply for divorce and free themselves from their husbands’ control, which is the norm in patriarchal societies such as Ethiopia. One would therefore have expected a relatively very high male/female suicide rate ratio among EI. However, this rate ratio was found to be similar to that among IB. This could be explained by the heavy pressure and mental cost that Ethiopian women pay for their relatively rapid adaptation to Israel, which often leads to a crisis in their relations with their husbands. In these situations, traditional leaders, the elderly (*Shmaglotch),* and priests (*Qessotch),* who had traditionally intervened in family and personal crises, lost their authority [37]. The high levels of family conflict among EI [38], as reflected by a higher divorce rate among EI than among the total Jewish population [14], might also explain that marriage was less protective against suicide for EI than for the general population, as was also reported among Ethiopian immigrants in Toronto [39]. The conflicts even reached physical violence as indicated by a high prevalence of intimate partner homicide and the fact that EI are significantly more prone to commit suicide after killing their partner than are the FSU and IB [40]. Suicidality among Ethiopians also appear to be more violent, the proportion of completed suicides being higher, as we can see from the fact that while suicide rates among EI are much higher than other populations, suicide attempt rates in this population were reported similar to those among the Jewish population without EI and FSUI immigrants, during all the years between 2006 and 2017 [17]. However, it should be noted that the lower suicide attempt rate may also be due to under-reporting of attempts, since EI have lower total rates of visits to hospital emergency departments [41].

A comparison of age-specific suicide rates revealed substantially higher rates for EI than for FSUI and IB for all age groups. Among FSUI, the elderly had the highest suicide rate, and among IB, the age-specific suicide rates regularly increased with age, similarly to the trend found worldwide [2]. This has been explained by factors such as age-related decline, loss of autonomy, and a higher frequency of depressive symptoms among the elderly [42]. Surprisingly, among EI, the suicide rates did not increase with age. Seeking an explanation, we considered calculating suicide rates by time since immigration, but this step was precluded due to data limitations. Different periods can be used as a proxy since the mean time since immigration increases over the years. So, we compared age-specific rates for EI during three periods: one closest to the large immigration waves, the second during the 10 subsequent years, and the third during the final years of the study period. The suicide rate of the youngest age group had the lowest suicide rate in the first study period (1985–2000); in the following years (2001–2010), the rate almost doubled, and then in the following years (2011–2017), decreased very steeply to a lower level than in the first years, but not significantly so. Apparently the youngest EI adjusted more quickly than the older immigrants to their new life in Israel. However, those having immigrated as children likely expected the same opportunities as the IB youth of their age, but substantial differences remained in education, employment, and socioeconomic levels [43]. Gaps between expectations and reality have been identified as a risk factor for suicide [42]. In the first decade of the twenty-first century which corresponds to our second period, a sharp increase in high-risk behavior has been observed among young EI [44], while the needed parental support was unmet [37]. The sharp decrease in the subsequent 7 years of the study (2011–2017) may be due to the observed rapid acculturation of the young EI [14].

Among the elder EI (ages 45–64), the highest suicide rate was recorded in the initial period of the study. Whereas most Ethiopians having arrived in Israel as adults came with minimal education and high illiteracy rates, by 2007, 15% had achieved post-secondary education in Israeli institutions [45]. This enhanced education level may have contributed to decreased suicide rates among older EI in 2001–2017, and it may reflect adjustment to their new country.

It is worthwhile noting that, in parallel to the worldwide decrease in suicide rate between 1990 and 2016 [46], a general decline in the rate of suicide mortality was also found in the three populations of our study, but our results are not related to this decrease, since it was the same for EI and IB, leaving EI with a much higher rate than IB during all the years of the study. However, we observed large fluctuations in suicide rates over the years among EI, whereas FSUI and IB presented only minimal fluctuations. Ethiopian immigration to Israel occurred in three waves, each under very different circumstances, and thus, the proportion of EI from each wave changed over the years. However, since a similar secular trend was found among immigrants for each wave, this cannot explain the substantial changes in suicide rates over the years among EI.

Events in Israel during the examined periods may offer explanations for the changes in suicide rates. The decrease in suicide rates between 1992 and 2001 may reflect the EI’s progressive integration, along with some improved understanding of EI culture and idioms of distress by Israeli officials and professionals [47]. As noted, for EI women, this decrease in suicide rates began earlier and was more pronounced. The steep increase in suicide rates between 2002 and 2006 may stem from a drastic nationwide reduction in the monthly family allowances granted by the National Insurance Institute and in other social welfare benefits during the years 2002–2005 [48]. In 2006 suicide rates began to decline, likely due to the Israeli economic recovery, a restoration of some of the social welfare allowances and the increase in support of EI by NGOs [49]. It is noteworthy that the changes in social welfare allowances appeared to be linked to greater changes in suicide rates among EI men than among EI women, perhaps indicating men’s greater stress tied to their burden of responsibility to provide for their families.

Thus, socioeconomic status (SES) appears to be a risk factor for suicide in fragile populations, such as EI, unlike FSUI and IB. This hypothesis is corroborated by the fact that, in our study, changes in the National Insurance Institute policies primarily affect EI rather than the other populations. Low education, unemployment, and poverty, which are dimensions of social exclusion, have been identified as risk factors for suicide mortality [9]. Whereas FSUI were typically highly professionally qualified, EI arrived generally unskilled. More than 40% of FSUI who arrived in Israel between 1990 and 2007 possessed college degrees, versus 3% of EI [50]. Most Ethiopian immigrants had previously lived as peasants and artisans and endured considerable difficulty integrating into Israel’s modern labor market, as indicated by a much higher rate of unemployment than among FSUJ [51]. Consequently, due to their low SES, EI tend to live in towns or neighborhoods having poor schools, limited employment opportunities [52], and significantly lower average household expenditures in comparison with non-Ethiopian Israeli Jews [53]; this disparity would then make any financial assistance more critical for EI.

It is noteworthy that in 2009 the Israeli government ministries operated a suicide prevention pilot program for EI and FSUI immigrants, for teens, and for seniors in Rehovot, Ramla, and Kfar Kana [54]. It evolved into a national program in December 2013 under the name of the National Program for Suicide Prevention, continuing to this day [55]. To date, no evaluation of the program has been published, and no sudden decrease in suicide rates is apparent from the program’s inception. Consequently, this program cannot explain the steady decrease in the EI suicide rate since 2007.

Anyhow, both genders and all ages are likely to have been engaged in the process of adjustment to Israel over time, as documented by the fact that suicide rates were the lowest in Period 3 and by a steady decrease since 2006.

### Strengths and limitations

The strengths of the study are that it is a national study encompassing entire populations and that data are related to an extensive period (33 years). A limitation of the study is that the examination of risk factors was limited to those derived from demographic data. A further study should explore other potential risk factors, such as time since immigration, SES, education, occupation, mental illness, and health service utilization.

## Conclusions and policy recommendations

The considerably higher suicide rate among EI than among FSUI during the 33 years of the study, which was not prevalent in the countries of origin, suggests the importance of the immigrants’ integration difficulties and their coping abilities. Among EI, an important aspect of these difficulties is ongoing family conflict [38, 56], which explains that marriage is less protective against suicide for EI than for other Israeli populations. The EI suicide rate decrease over time and at all ages since 2006, though slow, offers a ray of light––progressive integration appears to be occurring, especially among EI women. The fluctuations in EI suicide rates over the years in parallel to changes in social welfare allowances may indicate a high vulnerability of low-SES EI. Since the economic impact of the current COVID-19 pandemic is particularly sever among low-income populations [57], particular attention to EI is certainly needed.

Given the substantial difference between the two immigrant populations during all 33 years of the study, suicide prevention programs sensitive to the Ethiopian culture should be implemented. As shown in the present study, such programs should focus on EI, especially men confronting socioeconomic challenges (e.g., financial, employment, and housing problems [14, 52]) and high levels of family conflict [40]. These two groups are generally identified by HMOs and welfare services. Due to the low level of self-disclosure among EI [58], we suggest that the National Program for Suicide Prevention implement screening for suicide risk among members of those two groups within HMOs and welfare services. A brief and psychometrically validated screening instrument for suicide-risk assessment could be adopted, such as the Columbia-Suicide Severity Rating Scale [59], which non-clinicians can easily be trained to use. Those diagnosed as being at risk for suicide should be encouraged to participate in tailored workshops by mental health providers, trained in programs proved to be effective in preventing suicide. These interventions should aim to strengthen skills relating to coping with hardship [58] and legitimizing professional help-seeking [60], given that EI underuse community mental health services, especially for common mental health disorders like depression and anxiety [32]. Ethiopian community leaders and mental health professionals of Ethiopian origin could help promote EI participation in these programs.

## Data Availability

Basic data on suicide in immigrants and other groups in Israel is available in periodic publications of the Ministry of Health, and as a downloadable excel file. https://www.health.gov.il/UnitsOffice/HD/MTI/info/Pages/suicides.aspx: Detailed data used for analysis is not available due to privacy restrictions.
